# Paravertebral paraganglioma with spinal extension: a case report

**DOI:** 10.1186/s13256-023-03971-5

**Published:** 2023-06-08

**Authors:** K. Anavi, R. Daya, S. Daya, K. Purbhoo, C. Profyris, M. N. Mpanza, C. E. Nel, Z. Bayat

**Affiliations:** 1 Division of Endocrinology and Metabolism, Department of Internal Medicine, Helen Joseph Hospital, 1 Perth Road, Rossmore, Johannesburg, South Africa; 2 Division of Endocrinology and Metabolism, Department of Internal Medicine, Faculty of Health Sciences, School of Clinical Medicine, University of the Witwatersrand, Johannesburg, South Africa; 3 Department of Radiology, Helen Joseph Hospital, University of the Witwatersrand, Johannesburg, South Africa; 4 Department of Nuclear Medicine and Molecular Imaging, Chris Hani Baragwanath Academic Hospital and Charlotte Maxeke Johannesburg Academic Hospital, Faculty of Health Sciences, University of the Witwatersrand, Johannesburg, South Africa; 5 Department of Neurosurgery, Helen Joseph Hospital, University of the Witwatersrand, Johannesburg, South Africa; 6 Department of Anatomical Pathology, National Health Laboratory Services, University of the Witwatersrand, 7 York Road, Parktown, Johannesburg, South Africa

**Keywords:** Paraganglioma, Spinal tumor, Neuroendocrine tumor

## Abstract

**Background:**

Paragangliomas are rare neuroendocrine tumors. While paragangliomas of the spine are rare, those located in non-cauda equina areas with spinal canal extension are even rarer.

**Case presentation:**

We present a case of a 23-year-old female of African descent with a primary thoracic paraganglioma with intervertebral extension resulting in displacement and compression of the spinal cord and extensive local invasion of the surrounding structures. This paraganglioma was functional with typical symptoms of catecholamine excess. Despite the aggressive nature of the paraganglioma, the patient only had isolated sensory symptoms in the left shoulder. Adequate alpha and beta-blockade were instituted prior to her undergoing surgery with near-total resection and complete preserved neurology. There was no underlying pathogenic genetic mutation found.

**Conclusions:**

Even though rare, paraganglioma should be considered in the differential diagnosis of spinal tumors. Genetic testing should be performed in patients with paragangliomas. One should exercise extreme caution in treating such rare tumors that may cause neurological deficits and careful surgical planning should be undertaken to avoid possible catastrophic complications.

## Introduction

Pheochromocytomas (PCC) arise in the adrenal medulla [1]. When found outside the adrenal gland they are referred to as paragangliomas (PGL) [1]. PGLs are rare neuroendocrine tumors and can occur sporadically or can be grouped among hereditary pheochromocytomas–paraganglioma syndromes (PPGL). PGLs develop embryologically from neural crest cells and may have different areas of distribution in the body [1]. As paraganglia are clusters of non-neuronal cells of the autonomic nervous system, they may be derived from either sympathetic or parasympathetic ganglia [2]. PGLs may arise along the paravertebral axis from the base of the skull to the pelvis [1]. Their location within the body is variable and may be non-secretory.

The majority of parasympathetic PGLs are found in the head and neck region along the cranial nerve branches of the vagus and glossopharyngeal nerves [3]. In addition, they are often non-secretory [3]. PGLs that are found along the sympathetic chain are often within the abdominal region and are usually secretory [1]. They are specifically common in the para-aortic area near the junction of the inferior vena cava and left renal vein, as well as close to the organ of Zuckerkandl around the origin of the inferior mesenteric artery [4]. Few arise within the thoracic cavity and even fewer can occur within the spine [4]. Less frequent locations include the larynx, gallbladder, thyroid, prostate, mediastinum, and retroperitoneum [5, 6]. Spinal PGLs are exceedingly uncommon [7].

Primary PGLs of the central nervous system are extremely rare [8]. According to the World Health Organization (WHO) classification of tumors of the central nervous system, they are divided into cranial and paraspinal nerve tumors [8]. There have been more than 250 cases of spinal PGLs described, most of which are in the lumbosacral regions with a classic anatomical site of the cauda equina [9]. The majority of the spinal PGLS are noted to be nonfunctional and with extra-medullary location [9]. Limited data exists about cervical and thoracic site lesions making clinical management vague [8]. Importantly, few thoracic paraspinal PGLs are secretory with four distinct cases [10]. In a recent systematic review of primary PGLs of the spine, only 18 patients out of 334 cases had functional tumors and of those, 8 were in non-cauda equina locations [8].

We present a case of a young African female with a functional PGL occurring in the paravertebral space of the thoracic spine, with local invasion and displacement of the spinal cord.

## Case

A 23-year-old female of African descent, recently diagnosed with hypertension, was referred to the endocrine clinic for investigation. She had a background history of human immunodeficiency virus (HIV) infection with a recent absolute cluster of differentiation 4 (CD4) count of 390 cells. She was virally suppressed on a first-line fixed-dose combination treatment of tenofovir-lamivudine-dolutegravir. There was no other significant past medical, surgical, or family history of note. On further history, the patient reported paroxysmal episodes of headaches, palpitations, and sweating. She did not have any focal neurological symptoms. On physical examination, she had a tachycardia of 110 beats per minute and a sustained elevated blood pressure with an average of 160/90 mmHg. There were no palpable neck or abdominal masses or any skin lesions. The rest of her systemic examination was unremarkable.

On biochemical investigation, she had significantly elevated urine catecholamine metabolites with normetanephrine levels of 50,060 nmol/24 h (562–2129 nmol/24 h), (Table 1). Plasma metanephrines testing was not available in South Africa at the time. Chromogranin A was extremely high with a value of 554.9 ng/mL (< 101.9 ng/mL). In view of our patient being young and hypertensive with classic triad symptoms, in combination with the biochemical results, a PPGL was suspected.

**Table 1:** Summary of biochemical results

Parameter	Result	Reference range (units)
Preoperative urine normetanephrine	50,060	562–2129 nmol/24 hour
Postoperative urine normetanephrine	66,124	562–2129 nmol/24 hour
Urine normetanephrine: creatinine ratio	6843	50–204 nmol/mmol creat
Urine VMA: creatinine ratio	11.4	1.6–4.7 µmol/mmol creat
Urine HVA: creatinine ratio	1.8	2–6 µmol/mmol creat
Chromogranin A	554.9	< 101.9 ng/mL
Absolute CD4	390	332–1642 cells/µL
Viral load	Lower than detectable limit	50–10,000,000 copies/mL

CD4, cluster of differentiation 4; CGA, chromogranin A; HVA, homovanillic acid; VMA, vanillylmandelic acid

**Fig. 1:**
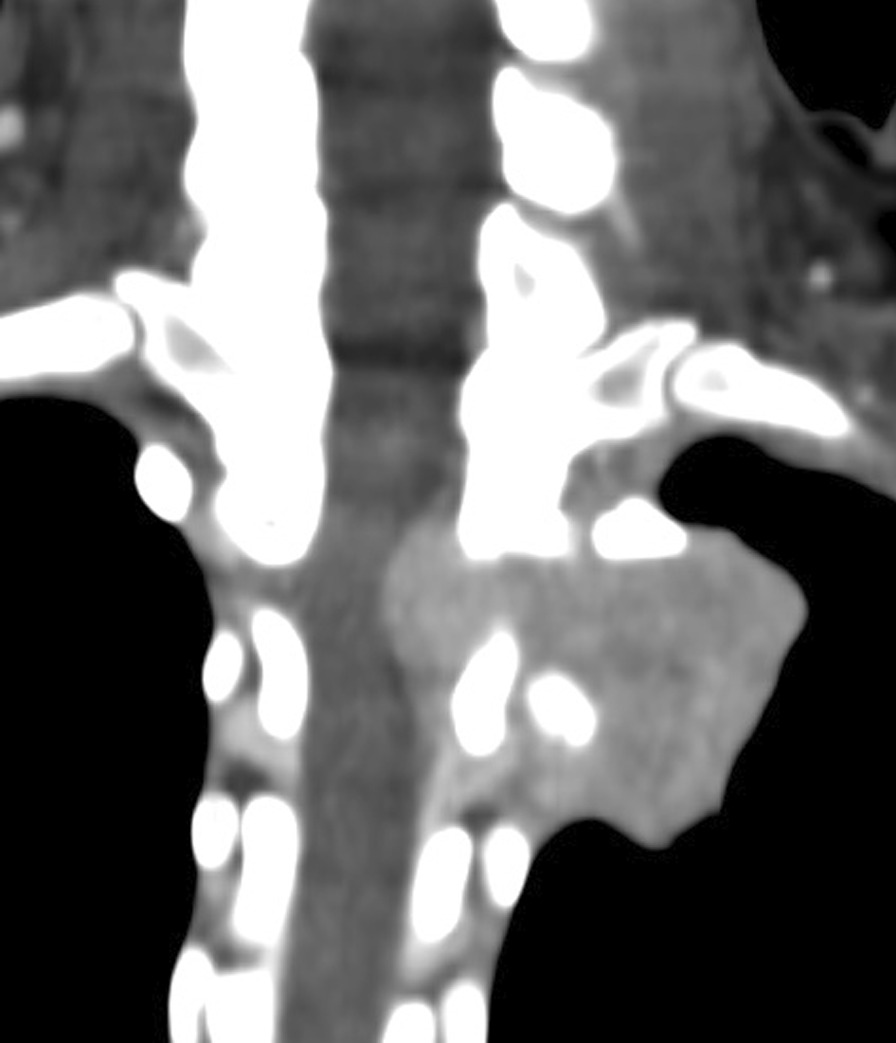
Contrast-enhanced coronal computed tomography (CT) imaging showing a large avidly enhancing left posterior mediastinal paraspinal soft tissue mass with medial extension into the second thoracic vertebral body and further extension into the intervertebral neural foramina, resulting in spinal cord abutment

**Fig. 2:**
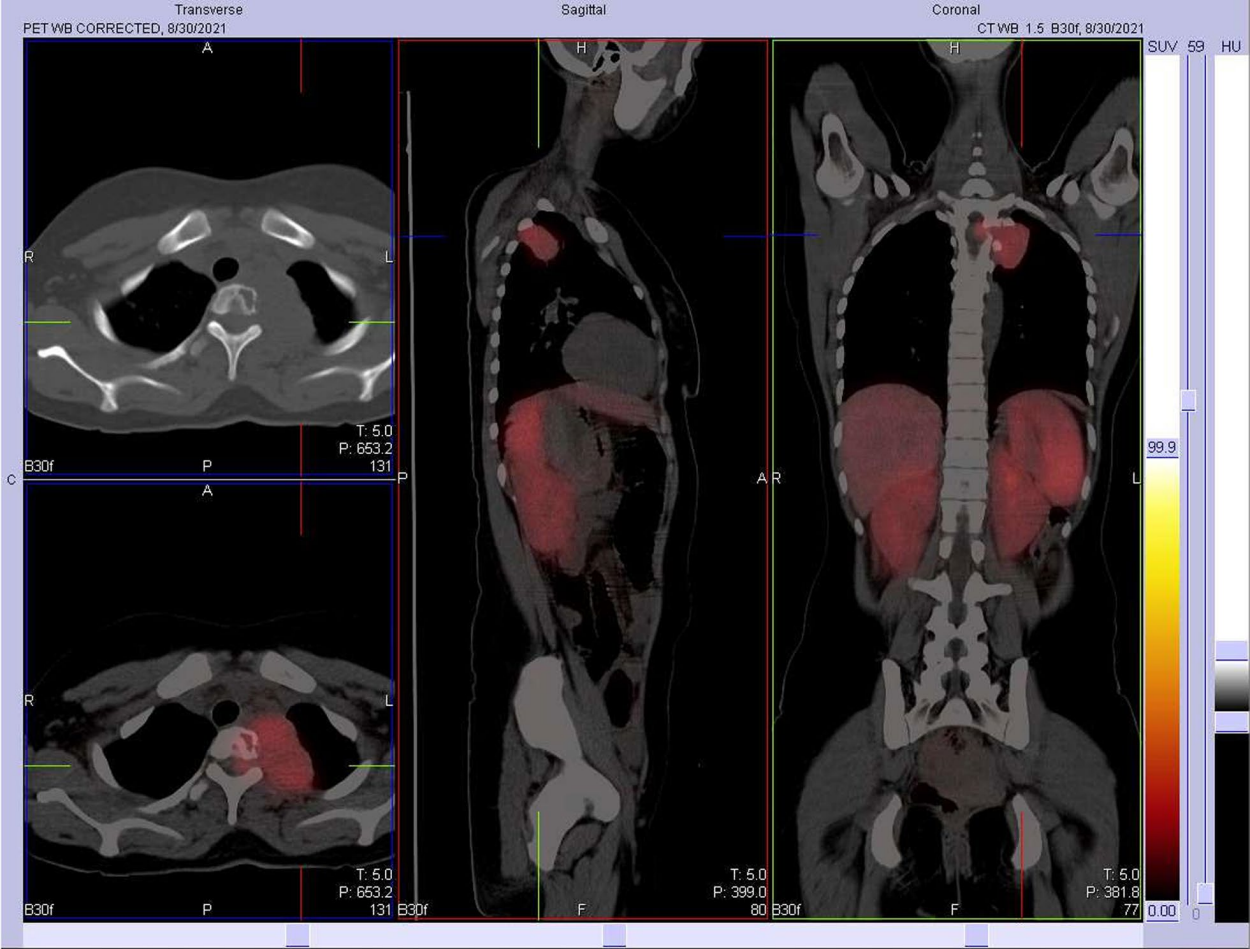
^68^Ga DOTATATE PET/CT Gallium Ga-68 DOTATATE scan showing receptor avid uptake in the left posterior mediastinal mass

Initial contrast-enhanced computed tomography (CT) of the abdomen was normal. Subsequent CT chest imaging showed a large avidly enhancing left posterior mediastinal T1–T3 paraspinal soft tissue mass abutting the apical-posterior segment of the left upper lobe of the lung. Medial extension into the second thoracic vertebral body, and even further extension into the intervertebral neural foramen, resulted in spinal cord abutment (Fig. 1). A ^68^Ga DOTATATE (68 Ga) 1,4,7,10-tetraazacyclododecane-1,4,7,10-tetraacetic acid (DOTA)–Octreotate scan showed DOTA receptor avid uptake in the left posterior mediastinal mass (Fig. 2). This scan supported the clinical diagnosis of a PGL, as the tracer has a high affinity for somatostatin receptors, which are overexpressed in neuroendocrine tumors. From the initial referral, it took approximately 6 months to come to a diagnosis.

**Fig. 3:**
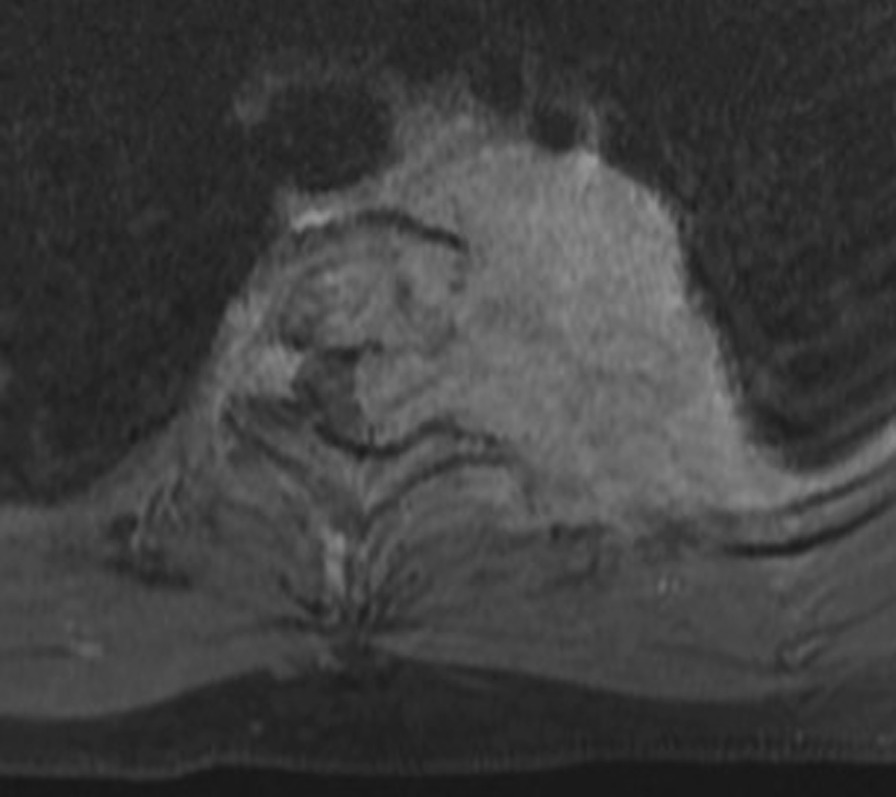
Magnetic resonance imaging (MRI) axial enhanced T1-weighted image at T2/T3 intervertebral level demonstrating avidly enhancing mass with neural foraminal extension and spinal cord displacement and compression. The mass is abutting the left subclavian artery and is eroding the left T2 transverse process, as well as the left third rib

Magnetic resonance imaging (MRI) demonstrated extension into the epidural space via the left neural foramina of T1/T2 and T2/T3 and with resultant displacement and compression of the spinal cord at the T2 level; however, no spinal cord edema was noted. There was additional infiltration of the posterior left parietal pleura with encasement of the left subclavian artery and abutment of the left common carotid artery, brachiocephalic trunk, and aortic arch posterior walls (Fig. 3). Despite the extent of the mass abutting the spinal cord and surrounding vessels, the patient did not have any obvious focal neurology. However, during her admission, she developed severe sudden shoulder pain and altered sensation of the left upper limb, which prompted the multidisciplinary team to aim for urgent surgical resection.

The patient was medically optimized with an alpha blocker (doxazosin) and a beta blocker (atenolol) prior to surgery. After 14 days of the alpha blockade and 72 h of beta blockade, our patient underwent emergency surgery, having a T2–T4 laminectomy and posterior resection of the PGL lesion. The surgery was uncomplicated and upon postoperative assessment, the patient had preserved neurology.

**Fig. 4:**
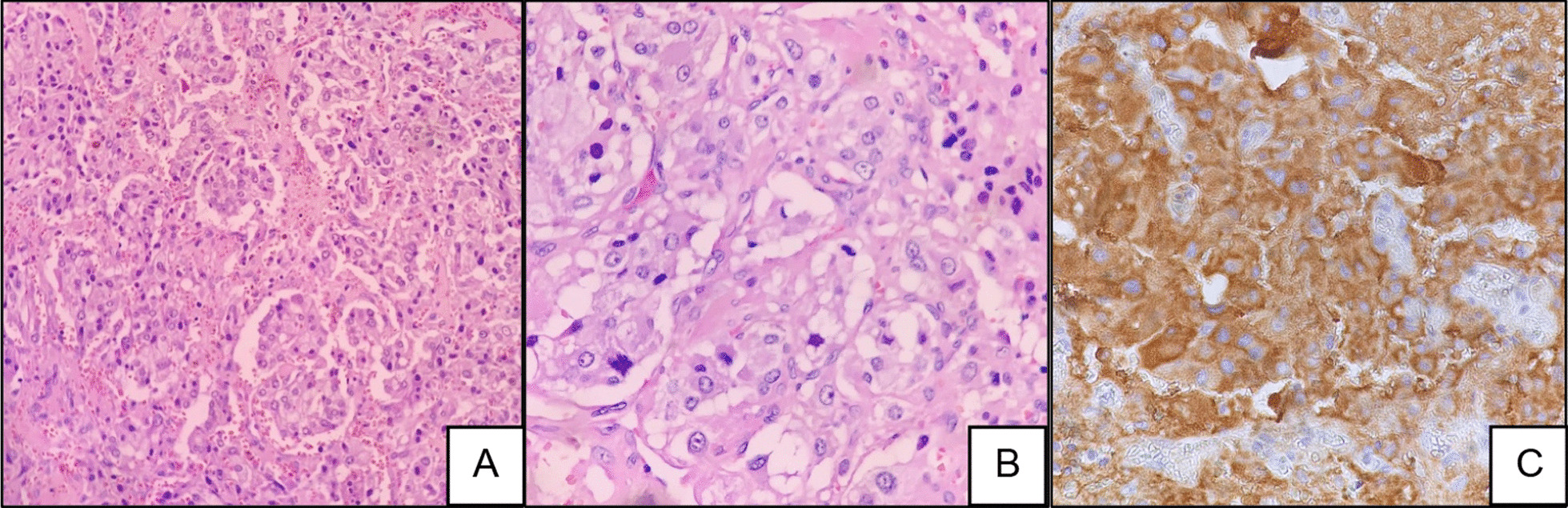
**A** Hematoxylin and eosin (H&E) stained section of the mass showing a characteristic nesting pattern (zellballen pattern) of cells (×200 magnification). **B** Hematoxylin and eosin (H&E) stained section of the mass showing a characteristic nesting pattern (zellballen pattern) of cells (×400 magnification). **C** Immunohistochemistry staining with chromogranin showing positive cytoplasmic staining (×400 magnification)

Histology confirmed the typical nested (zellballen) growth pattern of the tumor with invasion into the surrounding capsule and a focus of lymphovascular space invasion (Fig. 4A and B). Immunohistochemistry stains were diffusely positive for chromogranin (Fig. 4C). Histology and immunohistochemistry were in keeping with a well-differentiated PGL. The grading system for adrenal pheochromocytoma and paraganglioma (GAPP) was 1.

As genetic testing for PPGLs was not yet available in South Africa, a sample was sent to an internationally accredited commercial laboratory. The sample yielded no key pathogenic DNA mutation.

**Fig. 5:**
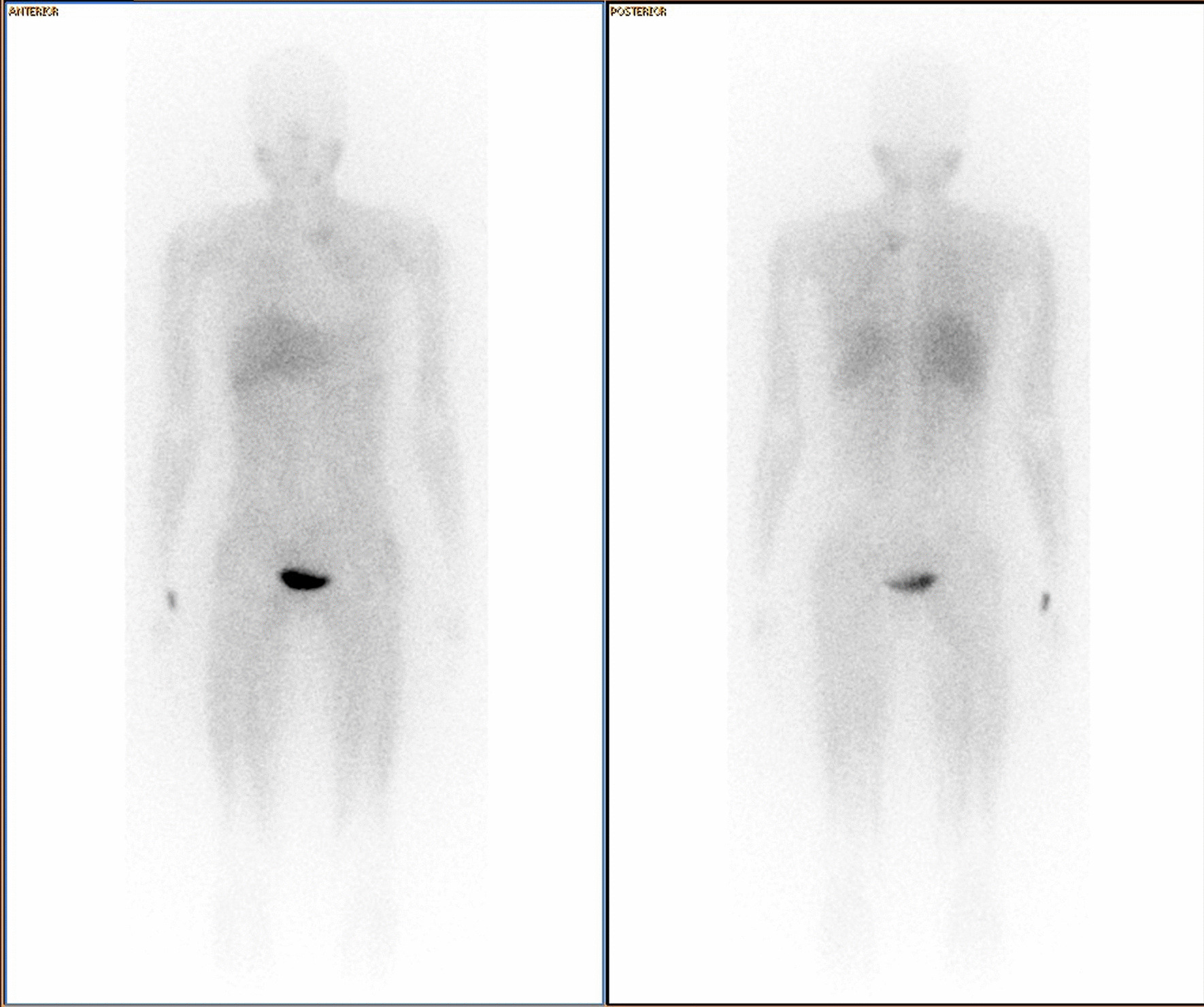
Single-photon emission computed tomography (SPECT/CT) images at 24 h showing uptake in the left paraspinal mass at T2 level, with involvement of T2 vertebra

**Fig. 6:**
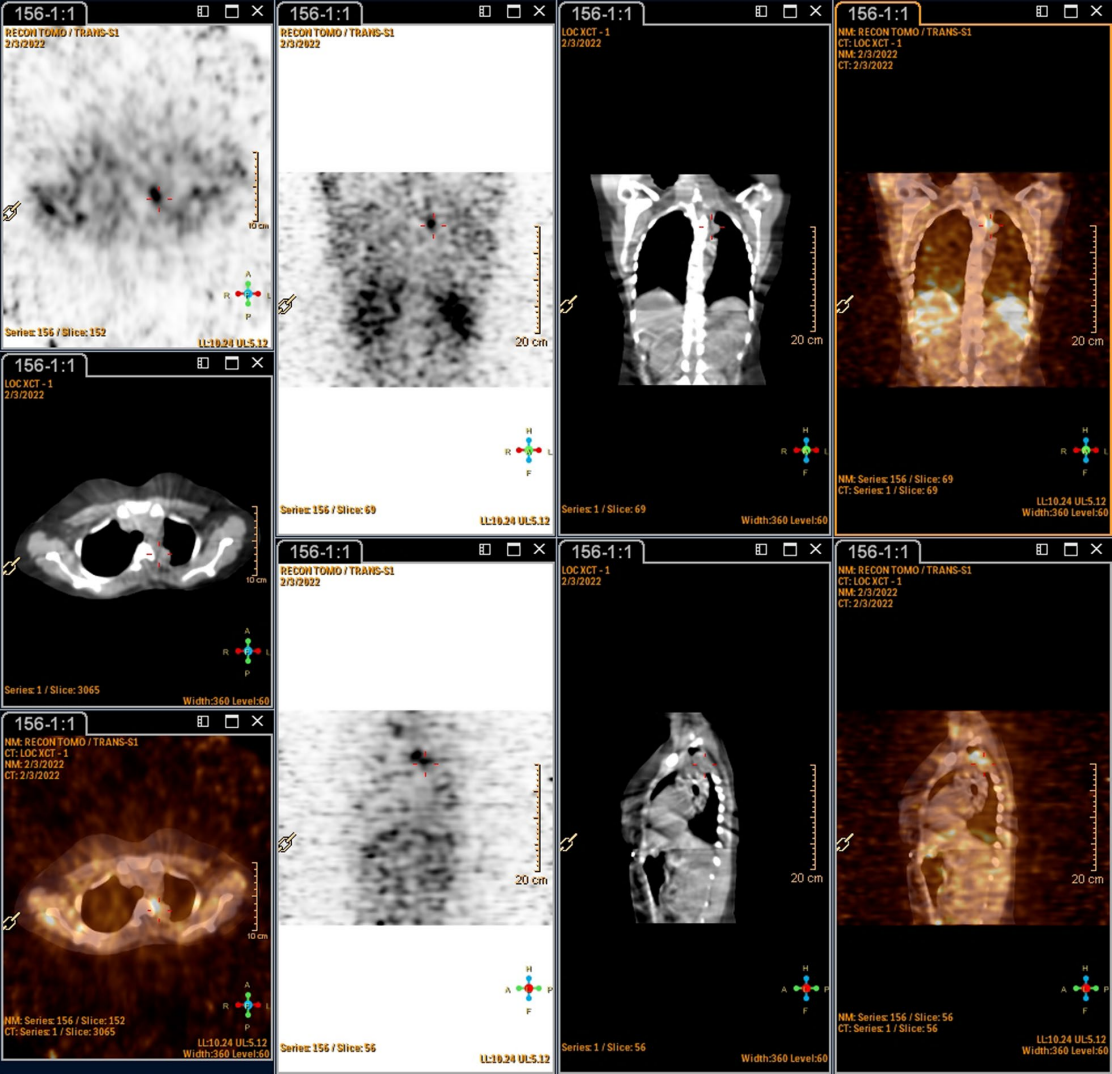
Whole body planar anterior and posterior MIBG images showing focal uptake in the left upper thorax, in the paraspinal region, corresponding to the mass seen on CT

Postoperatively she remained symptomatic; her blood pressure remained elevated. At 6 weeks, postoperation urine sampling and imaging were conducted. The 24-h urine metanephrines were still positive with a urine normetanephrine level of 66,124 nmol/24 h (562–2129 nmol/24 h). A postoperative meta-iodobenzylguanidine (MIBG) scan revealed that there was still avid disease in the left upper thoracic region, representing residual disease (Figs. 5 and 6). Medical therapy (alpha and beta blockade) was continued, and the patient was referred for radiation therapy. Unfortunately, thereafter the patient defaulted follow-up and unexpectedly demised.

## Discussion

Up to the 1970s, all cases of spinal PGLs were described as intradural lesions of the cauda equina [5]. In a recent systematic review of the literature, 334 cases were found, of which over 80% were cauda equina lesions, while the rest were situated in the thoracic, thoracolumbar, and cervical regions [8]. Extradural compression is rare and only a handful of cases have been mentioned in the literature [3]. Eighteen case reports of thoracic PGLs have been described and of those, the majority have been in the extradural space with associated spinal cord compression [11].

To the best of our knowledge, there are only four cases of functional thoracic PGLs in the literature, thus making our case the fifth [11]. Jeffs et al. described a middle-aged female with a functional extradural PGL at the T12 level with mild hyperesthesia in the right T12 dermatome that underwent successful resection [12]. Spector et al. described a secretory PGL in the right paraspinal region extending from T1–T5, with the extension into the right T3–T4 foramen involving the epidural space, but without spinal cord compression, in a pediatric patient [13]. Zileli et al. described a middle-aged male with a left paraspinal tumor at T10–11 level who underwent complete resection without any neurological complications [14]. Simpson et al. described a middle-aged female with a mediastinal mass with extension into the left T1–2 neural foramen and bony erosion of T1 and cord compression [11]. Three of the four cases had successful resection with no neurological complications, with one case having to have postoperative radiotherapy due to incomplete resection (refer to Table 2 for an overview).

**Table 2:** Overview of case reports

References	Age/gender	Presenting symptoms	Position	Spinal cord compression	Extent of resection	Recurrence	Other treatment
Jeffs et al. 2003	53F	Headache, facial flushes, and palpitations No neurological symptoms	Thoracic T12	Extradural with compression	T11-L1 laminectomy Partial T12 vertebrectomy No residual tumor	No	No
Spector et al. 2003	8 M	Headache, chest tightness	Thoracic T1–5	Extradural No compression	Right thoracotomy No residual tumor	No	No
Zileli et al. 2006	49 M	Facial flushing and palpitations Hypertension No neurological symptoms	Thoracic T10–11	Extradural No compression	T10 costo-transversectomy T10 formainectomy	No	No
Simpson et al. 2010	49F	Palpitations, diaphoresis, hypertension, progressive left arm pain	Thoracic T1–2	Extradural with compression	C7–T2 laminectomy, T1–2 transverse process and rib heads removed Residual tumor left	No	RT

*C* cervical vertebrae, *F* female, *L* lumbar vertebrae, *M* male, *RT* radiation therapy, *T* thoracic vertebrae

It is assumed that spinal PGLs originate from the sympathetic chain of the spinal cord or possibly from heterotopic neurons that are found along the branches proximal to the sympathetic trunk [5]. Thoracic PGLs seem to form a distinct group as compared to spinal PGLs of the cauda equina [6]. They are more commonly extradural, have spinal cord compression, and have a possible predilection to produce metastases [6]. Compared with other extraadrenal PGLs, the lower incidence of secretory lesions may be due to a multitude of reasons, including the rarity of spinal PGLs, incomplete investigation preoperatively due to prominent neurological symptoms, and under-reported nonspecific symptoms, such as palpitations and anxiety [8]. Some spinal PGLs may present with mechanical-related symptoms viz. back pain, spinal cord compression associated with sensory abnormalities, and bladder and bowel involvement [8].

Approximately a quarter of extraadrenal and mediastinal PGLs have a 30–40% association with genetic syndromes, and it is extremely important to have a high index of suspicion for mutations [15]. Usually, PGLs of the head and neck region are benign but approximately 20%–25% of those in the abdomen and mediastinum may be malignant and high recurrence rates are reported [1]. Therefore, long-term surveillance is of utmost importance. In addition, due to the high vascularity of PGLs, complete surgical excision may be difficult leading to higher chances of residual tumor [6]. Furthermore, there may be possible recurrence [6]. There also may be metastases that cannot be removed. PGLs, whether biochemically active or not, have an increased malignancy occurrence when compared with PCCs [16]. In a metaanalysis reviewing baseline characteristics and outcomes in metastatic PPGLs, the majority of metastatic PPGL tumors were functional and associated with catecholamine over secretion symptoms [19]. In our case, malignancy was suspected due to our patient’s young age and the aggressive nature of the mass; however, there were no other features of malignancy.

Previously, genetic testing was considered when there is a family history, young age, and presence of multiple lesions [15]. Currently, the Endocrine Society and the European Society of Endocrinology recommend that all patients with PGLs should have genetic testing, regardless of age or family history [17]. More than 20 genes have been identified (both germline and somatic mutations) and they have different impacts on the tumor development [17]. Some PGLs are directly associated with genetic syndromes with common mutations, including mutations involving the rearranged during transfection (RET), succinate dehydrogenase subunits (SDHx), von Hippel-Lindau (VHL) syndrome, etc. [15]. Germline mutations of the SDH family are most common, and subunit B-related tumors are associated with a high chance of malignancy with metastases [4]. The genetic etiology of PPGLs has a strong impact on subsequent management and surgical approach [17].

Imaging modalities usually confirm the diagnosis with a discoverable mass, and nuclear medicine scans are extremely helpful to detect whether the mass is active. Scintigraphy with ^123^I MIBG (iodine-123-metaiodobenzylguanidine) is the preferred functional imaging method and specific diagnostic technique, when DOTA is not available [18]. Based on a growing body of literature with consistent superiority of ^68^Ga-DOTA PET/CT in the detection of PPGLs, it has become the primary functional imaging technique for PPGLs [17].

Once a diagnosis is confirmed, total surgical excision of the tumor is the primary goal as this provides a better long-term outcome [4]. Prior to surgery, adequate medical therapy involving sequential alpha and then beta blockers are used to normalize the hemodynamics and minimize intraoperative complications related to blood pressure control [1]. Surgery in these cases requires a multidisciplinary team and careful management intraoperatively, especially at the time of tumor removal. Patients require postoperative support in an intensive care unit. Whilst complete surgical removal of a spinal PGLs is the goal, it may not always be possible due to the invasion of the tumor into surrounding anatomical structures such as blood vessels, neural tissue, and organs [18], such as in the case of our patient. In addition, PGLs are highly vascularized tumors, which makes it difficult to achieve total surgical resection.

Cases of recurrence, when the tumor was not totally resected, have been described in the literature [9]. Chemotherapy, radiotherapy, use of octreotide, and somatostatin are effective adjunctive treatments for PGLs; however, there is no evidence of their use specifically in spinal PGLs and their use is controversial [18]. The role of postoperative radiotherapy is usually considered if there has been incomplete tumor removal or complete resection of a malignant PGL, although the data is not certain [1]. Moreover, radiation therapy has dose-related effects on adjacent normal tissue [1]. Patients need to have life-long follow-up, especially if they have an underlying pathogenic genetic mutation.

## Conclusion

Spinal PGLs are exceedingly rare, and herein we present a case of a young female of African descent, with a functional thoracic PGL with spinal canal extension and compression. There was no underlying pathogenic genetic mutation found in the patient. It is important that the management of cases involve a multidisciplinary team. Even though rare, PGLs should be considered in the differential diagnosis of spinal tumors. Genetic testing should be undertaken in the investigation of patients with PGLs for pathologic mutations. Malignancy should be suspected, with multiple and/or unusually located lesions. One should exercise extreme caution in treating such rare lesions that cause neurological deficits and undertake careful surgical planning to avoid catastrophic complications.

## Data Availability

The data used during the current study are available from the corresponding author upon reasonable request.

## Abbreviations

CT: Computed tomography
CGA: Chromogranin A
Ga-68 DOTATATE: (^68^ Ga) 1,4,7,10-tetraazacyclododecane-1,4,7,10-tetraacetic acid (DOTA)–Octreotate
GAPP: Grading system for adrenal pheochromocytoma and paraganglioma
HIV: Human immunodeficiency virus
MIBG: Meta-iodobenzylguanidine
MRI: Magnetic resonance imaging
PET/CT: Positron emission tomography/computed tomography
PGL: Paraganglioma
PCC: Pheochromocytoma
PPGL: Pheochromocytoma–paraganglioma
WHO: World Health Organization
